# Effects of Zilpaterol Hydrochloride Supplementation on Growth Performance, Carcass Characteristics and Meat Quality for Steers Differing in Breed Type

**DOI:** 10.3390/ani14040607

**Published:** 2024-02-13

**Authors:** Jesse O. Fulton, Janna J. Block, Keith R. Underwood, Stacy M. S. Zuelly, Kenneth C. Olson, Amanda D. Blair

**Affiliations:** 1 Department of Animal Science, University of Nebraska Panhandle Research & Extension Center, 4502 Ave I, Scottsbluff, NE 69361, USA; 2 Department of Animal Science, South Dakota State University, Brookings, SD 57007, USA; 3 Department of Animal Sciences, Purdue University, West Lafayette, IN 47907, USA

**Keywords:** beef, beta-agonist, breed, carcass, meat quality, zilpaterol hydrochloride

## Abstract

**Simple Summary:**

The β–adrenergic agonist zilpaterol hydrochloride (ZH) enhances skeletal muscle growth in beef cattle, resulting in a shift in the composition of gain. This results in improvements in feedlot performance and carcass yield but has been reported to decrease marbling score and beef tenderness. British (B) and British × Continental (BC) cattle possess inherent differences in muscle and fat composition and may respond differently to the growth-promoting mechanism of ZH. Therefore, the objective of this study was to determine the effects of ZH on growth performance, carcass characteristics and meat quality for B and BC steers. Steers with B (*n* = 76) or BC (*n* = 57) backgrounds were assigned to a control (no ZH) or ZH-supplemented treatment group. Cumulative growth performance was evaluated. Steers were subjected to ultrasound immediately before ZH supplementation and following withdrawal of ZH to evaluate changes in body composition. After harvest, carcass data were recorded, and muscle samples were collected to determine impacts on meat quality. Inclusion of ZH largely did not impact composition of gain and meat quality characteristics of B and BC cattle. Supplementation with ZH did improve loin muscle area and yield grade for steers but increased Warner–Bratzler shear force, indicating tougher steaks.

**Abstract:**

To determine the effects of zilpaterol hydrochloride (ZH) on growth performance, carcass characteristics and meat quality for steers differing in breed type, steers with British (B; *n* = 76) or British × Continental (BC; *n* = 57) backgrounds were allocated to a randomized incomplete block design with a 2 × 2 treatment structure. Pens within each block × breed type were randomly assigned to either ZH (8.3 mg/kg of DM; fed for 20 d before slaughter, followed by a 3-day withdrawal) or control (CON; 0 mg/kg ZH). Steers were subjected to ultrasound immediately before ZH inclusion and following withdrawal to determine the influence of ZH on changes in longissimus muscle area (LMA), fat thickness and percent intramuscular fat (IMF). Carcass data were collected, and the longissimus lumborum was collected for analysis of tenderness, moisture percentage, crude fat content, collagen content, postmortem proteolysis and sensory attributes. The ZH × breed type interaction did not influence (*p* > 0.05) the feedlot performance, carcass or meat quality attribute traits evaluated, with the exception of moisture percentage. Responses among breed types were as expected for B vs. BC cattle types. Supplementation with ZH improved (*p* < 0.05) LMA and yield grade but increased Warner–Bratzler shear force.

## 1. Introduction

The inclusion of zilpaterol hydrochloride (ZH, Zilmax^®^, Merck Animal Health, Summit, NJ, USA) in beef finishing diets has shown dramatic effects on skeletal muscle growth, shifting the composition of gain from fat to lean and resulting in improvements in average daily gain (ADG) and gain-to-feed (G:F) efficiency in the feedlot as well as increases in dressing percentage, hot carcass weight (HCW) and the cutability of the carcass [1,2]. However, research has also revealed that this product has a negative impact on tenderness, which can lead to consumer dissatisfaction and potentially impact beef demand [3,4,5]. To optimize the use of ZH to balance both yield and palatability characteristics, it is necessary to understand how ZH mechanistically functions across varied groups of cattle, feeding strategies and management conditions.

British and British × Continental cattle have inherent differences in muscle and adipose composition that could result in different responses to ZH. Thus, it is necessary to determine how differing breed types respond to the growth-promoting mechanism of ZH. Furthermore, it is also important to determine the impact of this potentially divergent response to growth on tenderness, as the pathways through which this enhanced muscle growth is believed to be accomplished greatly affect postmortem aging [2,6], which impacts beef tenderness. Additionally, collagen content and quality are key regulators for beef tenderness [7] and could be affected by ZH due to the rapid increase in muscle growth from feeding the beta-agonist.

Much work has focused on the effects of feeding ZH to a single ‘type’ of cattle (e.g., [3,8,9,10]), while research comparing the impact of feeding ZH to divergent ‘breed types’ with the aim of optimizing management strategies is limited. Therefore, our hypothesis was that the inclusion of ZH would differentially impact the composition of gain in British versus British × Continental cattle, thereby influencing the mechanisms that regulate beef tenderness. The objective of this study was to determine the effect of ZH on growth performance, carcass characteristics and meat quality for cattle with variations in breed type.

## 2. Materials and Methods

### 2.1. Animals

The South Dakota State University (SDSU) Institutional Animal Care and Use Committee approved all procedures involving animals (Approval Number 13-028E). Cows at the SDSU Antelope Research station, with primarily Angus genetics, were artificially inseminated by 1 of 2 bulls. Bulls were either 100% Angus or 50% Angus × 50% Simmental from a common Angus sire. Clean-up bulls that were 100% Angus from the same common sire were used for 60 days post-artificial insemination. Therefore, all progeny were grandsons of a common sire that was a trait leader for carcass characteristics in the Angus breed. Steer progeny (*n* = 133) were transported as one group after weaning (average age at weaning = 184 d) to the University of Nebraska-Lincoln Panhandle Research and Extension Center Feedlot. All steers were fed a common 60% roughage and 40% concentrate [dry matter (DM) basis] backgrounding ration for 45 days prior to the start of the project. At the start of the trial, all steers were fed a 45% roughage and 55% concentrate (DM basis) ration and were stepped up using three rations over a 63-day period to reach the final ration of 16% roughage and 84% concentrate (DM basis; Table 1). All steers remained on this ration until marketed. All steers were implanted with 36 mg zeranol (Ralgro^®^, Merck Animal Health, Summit, NJ, USA) at day −2 of trial initiation. All steers were re-implanted at day 70 with 200 mg trenbolone acetate and 40 mg estradiol (Revalor^®^-XS, Merck Animal Health, Summit, NJ, USA).

### 2.2. Experimental Design and Treatments

Steers representing British (B, Angus sired, *n* = 76) and British × Continental (BC, Angus × Simmental sired, *n* = 57) breed types were allocated to a randomized incomplete block design with 4 blocks of B and 3 blocks of BC. Treatments were arranged as a 2 × 2 factorial with 2 sire breeds and finishing diets fed with (ZH) or without (CON) ZH (8.3 mg/kg of DM; Zilmax^®^, Merck Animal Health, Summit, NJ, USA) for 20 days prior to slaughter as per product label instructions. Initial body weight (BW) were 272.2 ± 18.9 kg and 277.3 ± 18.9 kg (least squares means ± SEM) for B and BC, respectively. Using initial BW as the blocking factor, steers were stratified by initial BW into 3 blocks of 4 pens and 1 block of 2 pens of B only (9 or 10 head per pen) with ZH treatment randomly assigned within each breed type × block combination. The experimental design resulted in 4 BW blocks (3 complete and 1 incomplete, with only 2 pens for B represented), 7 pen replicates per ZH treatment, 8 pen replicates for B, 6 pen replicates for BC, 4 pen replicates for each ZH × B treatment combination, and 3 pen replicates for each ZH × BC combination. Following a 3-day withdrawal from ZH as per product label instructions, steers were marketed in 2 groups (153 days and 182 days on feed) when their 12th rib backfat thickness was estimated to average 1.0 cm. All steers in blocks 3 and 4 (heaviest initial BW blocks) were marketed in the first group (*n* = 76), and all steers in blocks 1 and 2 (lightest initial BW blocks) were marketed in the second group (*n* = 57).

### 2.3. Ultrasound Measurements

Real-time ultrasound measurements were collected and interpreted by the National CUP lab (Ames, IA) to determine 12th rib subcutaneous fat thickness, percent intramuscular fat (IMF) and LM area (LMA) for each steer using an Aloka 500V instrument (Aloka, Wallingford, CT, USA) equipped with a 17 cm linear array and 3.5 MHz transducer. Initial ultrasound measurements were collected 4 days prior to ZH inclusion, and final measurements were collected on the morning of harvest. Body weights were also collected at these time points. Differences (final–initial) in fat thickness, IMF and LMA were calculated to evaluate changes in carcass composition during the ZH feeding period.

### 2.4. Carcass Data and Sample Collection

Final BW was measured when steers were subjected to ultrasound on the morning of slaughter (final BW was reduced by 4% to represent a standard shrink). Steers were transported approximately 198 km to a commercial packing plant, where they were slaughtered under standard procedures. Steers were tracked through the harvest floor to maintain animal identification. Hot carcass weight for each individual carcass was recorded. Longissimus muscle area, 12th rib backfat and percentage KPH were recorded by university-trained personnel. Marbling score and United States Department of Agriculture (USDA) Quality Grade were assigned by a USDA grader. Hot carcass weight, LMA, 12th rib backfat and KPH were then used to calculate USDA Yield Grade for each individual carcass [11]. 

Following carcass chilling (approximately 48 h), carcasses were ribbed between the 12th and 13th ribs on the right side and the exposed longissimus dorsi was allowed to bloom for approximately 30 min before objective color (L*, a* and b*) measurements were recorded. A Minolta colorimeter (model CR-310; Minolta Corp., Ramsey, MJ; 50 mm diameter measuring space and D65 illuminant) was used to obtain measurements from two locations on the right longissimus dorsi (medial and lateral) and averaged for each carcass. The right longissimus lumborum was collected from each carcass and transported under refrigeration (2.2 °C) to the SDSU Meat Laboratory in Brookings, SD. At approximately 72 h postmortem, samples were trimmed to 0.64 cm of external fat. The connective tissue, gluteus medius and multifidus dorsi were removed so that only the longissimus muscle remained. Beef samples were then immediately cut into 0.6 cm slices or 2.54 cm steaks. A 0.6 cm slice was removed from the anterior face of the longissimus lumborum, aged for 3 days and assigned for determination of moisture and crude fat percentage. Nine steaks (2.54 cm) were then fabricated with steaks 1, 3, 5, 7 and 9 being assigned for determination of Warner–Bratzler shear force (WBSF) and percent cook loss at 3, 7, 14, 21 and 28 days of aging, respectively. Steaks 2, 4 and 6 were utilized for sensory evaluation by a trained sensory panel at 3, 14 and 28 days of aging, respectively. Steak 9 was assigned for collagen evaluation and was aged for 14 days to determine collagen content and solubility. Following fabrication of steaks, the next five 0.6 cm slices were assigned for determination of troponin-T proteolysis at 3, 7, 14, 21 and 28 days of aging, respectively. All samples were vacuum-sealed and aged in the absence of light at 2–3 °C and were immediately frozen (−20 °C) after each specified aging period and checked regularly for seal integrity until thawed for specific analyses. 

### 2.5. Moisture and Crude Fat Percentage

Moisture percentage and ether extractable fat percentage for the longissimus muscle were determined according to procedures described by Mohrhauser et al. [12]. Steaks were thawed slightly and trimmed of external fat and additional muscles; then, samples were minced, immersed in liquid nitrogen and powdered using a commercial blender. Duplicate powdered samples (5 g) were weighed into dried tins, covered with dried filter papers and dried in an oven at 101 °C for 24 h. Dried samples were then reweighed after cooling. Moisture content was calculated as the difference between pre- and post-drying sample weights and expressed as a percentage of the pre-drying sample weight. Dried samples were extracted with petroleum ether in a side-arm Soxhlet extractor (Thermo Fischer Scientific, Rockville, MD, USA) for 60 h followed by drying at room temperature and subsequent drying in an oven at 101 °C for 24 h. Dried, extracted samples were cooled then reweighed. Crude fat was calculated as the difference between pre- and post-extraction sample weight and expressed as a percentage of the pre-extraction sample weight.

### 2.6. Warner–Bratzler Shear Force and Cook Loss

To determine objective tenderness, steaks were thawed for 24 h at 4 °C for shear force evaluation. All steaks were weighed prior to cooking on an electric clam-shell grill (George Forman Grilling Machine, Model GRV-120 GM, Lake Forest, IL, USA) to a target internal peak temperature of 71 °C. Peak internal cooked temperature measurements were recorded for each steak using a handheld thermometer (Thermoworks, Super-Fast^®^ Pocket Thermometer Model: RT600C, Lindon, UT, USA) placed near the geometric center of the steak. After cooking, all steaks were allowed to cool to room temperature before they were reweighed to determine cook loss. Cook loss was reported as a percentage of the raw weight using the following equation: [(raw weight − cooked weight)/raw weight] × 100. Cooked steaks were cooled for 24 h at 4 °C before removing 6 cores (1.27 cm in diameter) parallel to the muscle fiber orientation [13]. A single peak shear force measurement was obtained for each core using a Warner–Bratzler shear machine (G-R Electric Manufacturing Company, Manhattan, KS, USA). Shear force values of each core (*n* = 6 per steak) were averaged to determine the peak shear force value for each steak.

### 2.7. Collagen Content and Solubility

To determine if ZH differentially impacted growth and tenderness parameters for different breed types of cattle through alterations in connective tissue, 14-day aged steaks from 2 heads per pen (*n* = 28) closest to pen average for WBSF were selected and analyzed for collagen content and solubility. Total intramuscular collagen and percent soluble collagen of muscle samples were determined using a modified procedure of Hill et al. [14], as described by Gerrard et al. [15].

### 2.8. Postmortem Proteolysis

To determine if ZH differentially impacted growth and tenderness parameters for different breed types of cattle through changes in proteolysis, longissimus muscle samples were selected from slices that had been aged for 3, 14 or 21 days from 2 heads per pen closest to pen average for WBSF. Following the assigned aging period, each sample was frozen until preparation for SDS-PAGE and Western blotting. Immunoreactive intact troponin T (a classical marker of postmortem proteolysis) was identified and quantified as a measure of the extent of postmortem proteolysis as described by Mohrhauser et al. [16], with slight modifications. The abundance of troponin T is expressed relative to the abundance of actin and normalized back to two common molecular weight standards (Bio Rad #161-0374 Precision Plus Protein Dual Color Standards Bands 50 and 37 kD).

### 2.9. Trained Sensory Panel

Eight sensory panelists were trained to evaluate meat quality attributes for longissimus lumborum steaks according to the American Meat Science Association training guidelines appropriate for the study [13]. Steak samples were evaluated for juiciness (1 = extremely dry; 18 = extremely juicy), tenderness (1 = extremely tough; 18 = extremely tender) and beef flavor (1= extremely bland; 18 = extremely intense) on an eight-point hedonic scale. Steaks were cooked on an electric clamshell grill (George Forman Grilling Machine, Model GRV-120 GM, Lake Forest, IL, USA) to an internal temperature of 71 °C. After cooking, steaks were rested for five minutes and then cut into 2.5 × 1 × 1 cm samples. Two cubes were placed into a prelabeled plastic cup, covered with a plastic lid to retain heat and moisture and held in a warming oven (Metro HM2000, Wilkes-Barre, PA, USA) at 60 °C until served. Evaluations were performed according to American Meat Science Association guidelines [13]. Sample evaluations were alternated by treatment to reduce first and last order bias. Samples were served to panelists in a randomized fashion, and panelists were isolated in individual booths, under red lights to limit observation of visual differences.

### 2.10. Statistical Analysis

Continuous response variables, including growth, measured carcass traits and meat quality measurements were analyzed in a 2 × 2 factorial treatment structure in a randomized incomplete block design using the mixed procedure of SAS version 9.3 (SAS Institute, Cary, NC, USA). Pens served as the experimental units and were included as a random effect. Breed type, ZH treatment and their interaction were included as fixed effects. The Kenward–Roger option was used to calculate denominator degrees of freedom. Peak internal cooked temperature was included as a covariate for WBSF, cook loss and sensory scores. Least squares means were calculated and separated by F-tests for fixed effects. The breed type × ZH interaction was not significant (*p* > 0.05) for any response variable except steak moisture percentage (*p* = 0.0496). For this interaction, lsmeans were calculated and separated using least significant differences (PDIFF) with a Tukey adjustment. As the Quality and Yield Grade classifications for each carcass conform to binomial distributions, the proportions (number graded in the class divided by number in the pen) for carcasses in each grade classification were analyzed as binomial distributions in the GLIMMIX procedure of SAS using the same model as above. Proteolysis and WBSF values were analyzed using day of aging as a repeated measures using the mixed procedure of SAS. Least square means for the aging effect were calculated and separated using the Tukey adjustment. The aging effect did not interact with treatments for any response variable (*p* > 0.05). Reponses were considered significant at *p* ≤ 0.05, and tendencies were considered at *p* > 0.05 to *p* ≤ 0.10.

## 3. Results

### 3.1. Feedlot Performance and Carcass Traits

The ZH × breed type interaction did not influence (*p* > 0.05) any of the feedlot performance or carcass traits evaluated in this study (Table 2).

Breed type did not affect (*p* > 0.05) final BW, cumulative ADG, DMI or G:F for steers (Table 2). The changes in ultrasound fat thickness, LMA, percent IMF and ADG during the ZH feeding period were not different (*p* > 0.05) between the cattle breed types investigated in this study (Table 2). Carcass evaluation revealed no differences (*p* > 0.05) in HCW or fat thickness between breed types. However, BC had a larger (*p* < 0.05) LMA and improved (*p* < 0.05) Yield Grade compared with B carcasses. The marbling score was increased (*p* < 0.001) in B carcasses compared with BC carcasses (Table 2). British × Continental steers produced a greater proportion (*p* < 0.05) of Yield Grade 2 and fewer Yield Grade 3 (*p* < 0.05) carcasses than B steers (Table 2). However, a greater proportion (*p* < 0.01) of B carcasses were classified as Upper 2/3 Choice. There was no difference between breed types for the number of carcasses graded as Select. However, there was tendency for an increase (*p* < 0.10) in the proportion of BC carcasses classified in the Lower 1/3 Choice grade compared with B carcasses (Table 2). 

Supplementation with ZH did not affect (*p* > 0.05) final BW, overall ADG or DMI over the entire feeding period, but tended to improve (*p* < 0.10) overall G:F (Table 2). However, ADG during the 20 d ZH feeding period was improved (*p* < 0.05) by ZH supplementation. The differences between ultrasound measurements 4 days prior to ZH supplementation and on the day of slaughter revealed ZH-treated cattle did not affect (*p* < 0.05) ultrasound measures for LMA or fat thickness compared with CON, while ZH reduced (*p* < 0.05) ultrasound measures for IMF percentage compared to CON cattle during the supplementation period (Table 2). Supplementation with ZH did not influence HCW (*p* > 0.05). However, the carcasses of ZH-treated steers had greater (*p* < 0.001) LMA and improved (*p* < 0.05) Yield Grades. Despite the greater accretion of IMF during the ZH feeding period, CON carcasses had similar (*p* > 0.05) marbling scores to ZH carcasses. The trend for carcass fat thickness was similar (*p* > 0.05), in agreement with similar changes between treatments in ultrasound fat thickness during the ZH feeding period (Table 2). Additionally, steers supplemented with ZH tended to produce a greater (*p* < 0.10) percentage of Yield Grade 2 carcasses and a lower (*p* < 0.10) percentage of Yield Grade 3 carcasses than CON steers (Table 2). Supplementation with ZH did not affect (*p* > 0.10) the distribution of Quality Grades compared with CON carcasses. 

### 3.2. Meat Quality Traits

Steaks from B carcasses had greater Minolta L* and b* color values (*p* < 0.05) compared to BC steaks, while a* values did not differ (*p* > 0.05) between breed types (Table 3). Steaks from BC carcasses had decreased intramuscular fat percentage (*p* < 0.001) compared to steaks from B carcasses (Table 3). An interaction was observed for breed type × supplementation treatment (*p* < 0.05) for steak moisture percentage. Steaks from the BC × CON treatment had greater (*p* < 0.05) moisture percentages (71.27 ± 0.446%) than steaks from B cattle in either ZH treatment (69.27 ± 0.422% and 70.09 ± 0.422% for B × CON and B × ZH, respectively), with BC × ZH having intermediate and similar (*p* > 0.05) moisture percentages to all other treatments (71.07 ± 0.446%). Breed type did not influence (*p* > 0.05) WBSF values; however, there was a tendency (*p* < 0.10) for increased cook loss for B steaks. Collagen content and solubility did not differ (*p* > 0.05) among breed types. British steers had less (*p* < 0.05) intact troponin T compared to BC steers (Table 3). No differences were detected (*p* > 0.05) among breed types for sensory panel ratings for tenderness, juiciness, beef flavor, off flavor or overall acceptability (Table 3).

Supplementing with ZH did not influence (*p* > 0.05) L* or a* but tended (*p* = 0.10) to lower b* (Table 3). Steaks from cattle supplemented with ZH did not differ (*p* > 0.05) in moisture or fat percentages. Supplementing cattle with ZH increased WBSF values (*p* < 0.001) and percent cook loss (*p* < 0.001). Zilpaterol hydrochloride supplementation did not influence (*p* > 0.05) collagen content and solubility or the degradation of troponin T. (Table 3). Sensory panel scores indicated that tenderness, juiciness and overall acceptability were reduced (*p* < 0.001), and beef flavor and off flavor tended (*p* < 0.10) to be reduced by ZH supplementation.

Aging did not interact (*p* > 0.10) with treatments for WBSF and intact troponin T percentage. However, as expected, WBSF was reduced (*p* < 0.001) as steaks aged (4.55, 3.95, 3.37, 3.16 and 3.04 ± 0.097 kg for 3, 7, 14, 21 and 28 days of aging, respectively). In addition, intact troponin T percent was reduced (*p* < 0.001) with postmortem aging (0.850, 0.342 and 0.221 ± 0.11% at 3, 14 and 21 days, respectively).

## 4. Discussion

Beta-adrenergic agonists commonly elicit compositional changes by increasing muscle synthesis and decreasing adiposity for growing animals [2,17]. Previous research has investigated the impact of ZH on performance and carcass characteristics within similar breed types of cattle such as calf-fed Holsteins [18,19], however it is unknown whether cattle of divergent genetic backgrounds will respond differently to ZH. Therefore, the objective of this study was to determine whether cattle from different breed types would have a differential response to ZH supplementation.

The general lack of breed type × ZH treatment interactions suggested that the breed types evaluated in this study did not differentially respond to ZH supplementation. Gruber et al. [20] investigated the effects of ractopamine hydrochloride on feedlot steers of varying genetic backgrounds and reported no interaction between treatment and breed type. Ractopamine hydrochloride functions to increase protein synthesis, while ZH has been shown to both increase protein synthesis and decrease degradation, resulting in increased LMA, decreased fat thickness and higher-yielding carcasses [8]. Although the response to the beta-agonist’s main effect differed between the study of Gruber et al. [20] and the current study, the lack of interaction in both studies indicated that steers from different genetic backgrounds respond similarly to beta-agonists, regardless of the mode(s) of action for the specific beta-agonist. McEvers et al. [21] sorted cattle into tenderness genotype groups based on a commercial DNA panel and also detected no ZH × genotype interactions, further supporting the general lack of differential response to beta-agonist supplementation found in the current study. The only breed type × ZH supplementation interaction observed in the current study was in relation to the moisture content of steaks. However, differences among these moisture values are likely not detectable by consumers.

The response by breed types was as expected. Gruber et al. [20] reported similar feedlot performance responses as the current study with British × Continental cattle displaying similar ADG, DMI and G:F results to British cattle when fed diets with or without the beta-agonist ractopamine. However, in Gruber et al. [20], the Continental cross cattle had greater initial and final BWs, which was not the case in the current study which evaluated the beta-agonist zilpaterol. This difference between studies for BW was further observed for HCW, which was similar among the breed types herein but heavier in Gruber et al. [20]. Gruber et al. [20] and the current study agreed that LMA was greater for British × Continental cattle, which lead to lower USDA Yield Grades, while marbling score was lower for British × Continental cattle. However, Gruber et al. [20] reported that backfat thickness was reduced in Continental crosses, while it was similar to British cattle in the current study. Although, there were slight shifts in percentages of carcasses in each USDA Yield and Quality Grade class, both studies displayed agreement that the incorporation of Continental breeds reduced USDA Yield (a lower grade classification equates to a higher lean yield in carcasses) and Quality Grades. Both studies were in agreement that WBSF was not affected by breed composition. However, sensory panel results differed. The current study indicated that panelists did not detect differences in sensory traits among breed types, while Gruber et al. [22] reported that panelists found British cattle to have improved tenderness, juiciness and beef flavor, with a lower off flavor than British × Continental crosses. Differences among studies may be related to the actual British or Continental breeds used in each study. Overall, the results indicate that the utilization of Continental breeds results in higher-yielding carcasses but reductions in marbling.

In this study, ADG was increased by ZH supplementation during the ZH feeding period, but this did not contribute to an overall improvement in ADG for the entire finishing period. Additionally, final BW and overall DMI were not affected by ZH supplementation, although G:F tended to be improved. Avendaño-Reyes et al. [23] also reported that the inclusion of ZH did not influence DMI but did improve G:F. The inclusion of ZH did not influence HCW in this study, which is similar to the findings of Van Bibber-Krueger et al. [24] and Bloomberg et al. [25]. In contrast, others [8,9,19,21] have reported that feeding ZH increased HCW. The increases in LMA and decreases in Yield Grade by feeding ZH were in agreement with others [8,9,21]. However, the effect of ZH on fat deposition varied among studies. Similar to this study, no difference in fat thickness was reported by McEvers et al. [21], while the fat thickness was reduced by ZH in reports by Scramlin et al. [8] and Rathmann et al. [9]. Furthermore, the marbling score was reduced by ZH in some reports [9,21] but unchanged in others [8], including in the current study. Additionally, the shift to leaner Yield Grades in response to ZH supplementation as found in the current study has been reported by others [9,21]. Similar to this study, others have reported that ZH supplementation did not influence objective color evaluation [25,26]. In agreement with the current study, the Warner–Bratzler shear force has been consistently increased by ZH supplementation [8,10], indicating a toughening effect. However, collagen content and degradation of troponin-T did not differ among treatments, suggesting that differences in tenderness are related to other mechanisms or the degradation of other proteins. Rathmann et al. [10] also reported no differences in collagen content in response to ZH supplementation, while Kellermeier et al. [4] reported no effect of ZH on degradation of desmin. Also in agreement with this study, Garmyn et al. [27] indicated that sensory panels detected decreased tenderness, beef flavor and overall liking in response to ZH supplementation. The only disagreement was that juiciness was decreased in the current study, but no difference in juiciness was detected by the sensory panel reported by Garmyn et al. [27]. The reduction in juiciness detected by the sensory panel for steaks from ZH supplemented cattle in the current study could be related to the increased cook loss detected in the ZH samples. 

## 5. Conclusions

We reject our hypothesis that the inclusion of ZH would differentially impact the composition of gain and meat quality characteristics in British versus British × Continental cattle. This study suggests a consistent response to ZH across the breed types of cattle considered herein. This may allow for the targeted use of ZH to influence economically important carcass traits in response to beef market signals. For example, ZH supplementation may be warranted when market signals demand leaner, higher-yielding carcasses. Conversely, marketing channels that value consumer palatability may consider limiting ZH utilization due to negative effects on tenderness and other sensory traits. However, further confirmation of this effect across additional breeds would be important before industry adoption. 

## Figures and Tables

**Table 1:** Finishing diet composition.

Ingredient	Diet DM, %
Dry-rolled corn	64.0
Wet distillers grains with solubles	15.0
Corn silage	15.0
Liquid supplement ^1,2^	6.0
Nutrient composition, %	
Starch	51.40
NDF	15.46
CP	12.96
Fat	3.44
Ca	0.60
K	0.55
P	0.34
Mg	0.15
S	0.15

^1^ Liquid supplement contained 0.6% urea, 1.6% Ca, 0.3% salt, 0.02% potassium chloride; supplement also formulated to provide a dietary DM inclusion of 0.3% salt, 60 mg/kg of Fe, 40 mg/kg of Mg, 25 mg/kg of Mn, 10 mg/kg of Cu, 1 mg/kg of I, 0.15 mg/kg of Se, 1.5 IU/g of vitamin A, 0.15 IU of vitamin D, 8.81 IU/kg of vitamin E. ^2^ Rumensin (30 g/ton) and Tylan (9 g/ton) were added via micromachine.

**Table 2:** Least square means for performance and carcass traits as affected by main effects of breed type ^1^ and zilpaterol hydrochloride (ZH) ^2^ supplementation.

	Breed Type				ZH, mg/kg of DM				ZH × Breed
Item	B	BC	SEM ^3^	*p* > F ^4^	0	8.3	SEM ^3^	*p* > F ^4^	*p* > F ^4,5^
Final body weight, kg	580	588	14.65	0.453	578	590	14.03	0.282	0.703
Cumulative average daily gain, kg	1.76	1.79	0.04	0.521	1.75	1.81	0.03	0.222	0.543
Dry matter intake, kg·hd^−1^·d^−1^	10.0	10.1	0.58	0.574	10.1	10.0	0.58	0.423	0.143
Gain:Feed	0.18	0.18	0.02	0.831	0.17	0.18	0.02	0.093	0.371
ZH Supplementation Period									
Fat thickness change ^6^, cm	0.10	0.07	0.04	0.429	0.11	0.07	0.04	0.330	0.263
Longissimus area change ^6^, cm^2^	2.85	1.58	1.13	0.279	1.53	2.89	1.04	0.221	0.399
Intramuscular fat change ^6^, %	0.26	0.39	0.20	0.449	0.59	0.06	0.19	0.012	0.196
Period average daily gain, kg	1.04	1.12	0.10	0.556	0.89	1.27	0.10	0.020	0.328
Hot carcass weight, kg	356	362	9.77	0.461	352	366	9.20	0.102	0.768
Fat thickness, cm	1.46	1.30	0.08	0.178	1.42	1.34	0.07	0.433	0.899
Longissimus muscle area, cm^2^	88.1	92.0	2.26	0.028	86.3	93.9	2.18	<0.001	0.499
Yield grade ^7^	2.94	2.62	0.10	0.033	2.95	2.60	0.09	0.023	0.875
Marbling score ^8^	588	488	25.9	<0.001	541	531	24.9	0.575	0.851
USDA Yield Grade ^7^									
Yield Grade 2, %	32.4	57.4	7.87	0.044	34.9	54.6	7.66	0.086	0.714
Yield Grade 3, %	68.7	40.2	8.77	0.019	65.0	44.3	8.47	0.059	0.616
USDA Quality Grade									
Prime, %	17.3	11.1	7.41	0.650	14.6	13.3	7.11	0.918	0.918
Upper 2/3 choice, %	68.4	33.1	6.28	0.006	51.5	50.2	6.87	0.895	0.355
Lower 1/3 choice, %	17.6	36.8	6.44	0.076	21.9	30.7	6.34	0.356	0.911
Select, %	15.1	12.7	7.03	0.821	18.3	10.4	7.62	0.517	0.759

^1^ Breed type was British (B) or British × Continental (BC); ^2^ Zilpaterol hydrochloride was fed at 0 (CON) or 8.3 mg/kg (ZH) of DM during the final 20 d of the finishing period; ^3^ Standard error of the mean; ^4^ Probability of a greater F; ^5^ The breed type × ZH interaction did not affect (*p* > 0.05) any traits; ^6^ Change in ultrasound backfat thickness, LM area and intramuscular fat during the 20 day ZH feeding period; ^7^ Lower USDA yield grade represents a leaner carcass; ^8^ 400 = Slight^0^; 500 = Small^0^; 600 = Modest^0^.

**Table 3:** Least square means for meat quality as affected by main effects of breed type ^1^ and zilpaterol hydrochloride (ZH) ^2^ supplementation.

Item	Breed Type				ZH, mg/kg of DM				ZH × Breed
	B	BC	SEM ^3^	*p* > F ^4^	0	8.3	SEM ^3^	*p* > F ^4^	*p* > F ^4,5^
Minolta color value									
L*	44.6	42.6	0.41	0.004	43.7	43.6	0.35	0.798	0.296
a*	23.0	22.7	0.44	0.134	23.1	22.7	0.43	0.102	0.478
b*	10.3	9.7	0.31	0.015	10.2	9.8	0.30	0.067	0.467
Intramuscular fat content, %	7.60	5.47	0.49	<0.001	6.88	6.18	0.46	0.109	0.142
Warner–Bratzler shear force, kg	3.57	3.66	0.07	0.281	3.04	4.19	0.06	<0.001	0.089
Cook loss, %	21.8	21.3	0.19	0.058	21.1	22.0	0.18	<0.001	0.651
Total collagen content, mg/g ^6^	4.5	4.3	0.23	0.558	4.5	4.3	0.20	0.310	0.788
Soluble collagen content, % ^7^	17.8	16.3	0.70	0.313	16.8	17.4	0.70	0.809	0.893
Intact troponin T, % ^8^	0.37	0.58	0.07	0.032	0.42	0.53	0.07	0.232	0.257
Sensory panel scores ^9^									
Tenderness	5.5	5.4	0.02	0.719	6.3	4.6	0.22	<0.001	0.784
Juiciness	5.1	4.7	0.02	0.117	5.5	4.4	0.20	<0.001	0.192
Beef flavor	5.5	5.2	0.02	0.216	5.5	5.1	0.16	0.079	0.923
Off flavor	7.7	7.6	0.01	0.399	7.8	7.6	0.11	0.091	0.990
Overall Acceptability	5.5	5.2	0.02	0.355	6.1	4.6	0.19	<0.001	0.099

^1^ Breed type was British (B) or British × Continental (BC); ^2^ Zilpaterol hydrochloride was fed at 0 (CON) or 8.3 mg/kg (ZH) of DM during the final 20 d of the finishing period; ^3^ Standard error of the mean; ^4^ Probability of a greater F; ^5^ The breed type × ZH interaction did not affect (*p* > 0.05) any traits; ^6^ Calculated as mg collagen/g wet meat weight; ^7^ Calculated as a percent of total collagen; ^8^ Intact Troponin T expressed relative to the abundance of actin and normalized back to two molecular weight standards; ^9^ Sensory scores using an eight-point hedonic scale for tenderness (8 = extremely tender to 1 = extremely tough), juiciness (8 = extremely juicy to 1 = extremely dry), beef flavor (8 = extremely intense to 1 = non-detectable), off flavor (8 = non-detectable to 1 = extremely intense), overall acceptability (8 = extremely acceptable to 1 = extremely unacceptable).

## Data Availability

The data presented in this study are available on request from the corresponding author.

## References

[1] Delmore R.J., Hodgen J.M., Johnson B.J. (2010). Perspectives on the application of zilpaterol hydrochloride in the United States beef industry. J. Anim. Sci..

[2] Johnson B.J., Smith S.B., Chung K.Y. (2014). Historical overview of the effect of β-adrenergic agonists on beef cattle production. Asian-Aust. J. Anim. Sci..

[3] Holmer S.F., Fernandez-Duenas D.M., Scramlin S.M., Souza C.M., Boler D.D., McKeith F.K., Killefer J., Delmore R.J., Beckett J.L., Lawrence T.E. (2009). The effect of zilpaterol hydrochloride on meat quality of calf-fed Holstein steers. J. Anim. Sci..

[4] Kellermeier J.D., Tittor A.W., Brooks J.C., Galyean M.L., Yates D.A., Hutcheson J.P., Nichols W.T., Streeter M.N., Johnson B.J., Miller M.F. (2009). Effects of zilpaterol hydrochloride with or without an estrogen-trenbolone acetate terminal implant on carcass traits, retail cutout, tenderness, and muscle fiber diameter in finishing steers. J. Anim. Sci..

[5] Leheska J.M., Montgomery J.L., Krehbiel C.R., Yates D.A., Hutcheson J.P., Nichols W.T., Streeter M.N., Blanton J.R., Miller M.F. (2009). Dietary zilpaterol hydrochloride. II. Carcass composition and meat palatability of beef cattle. J. Anim. Sci..

[6] Koohmaraie M., Whipple G., Kretchmar D.H., Crouse J.D., Mersmann D.H. (1991). Postmortem proteolysis in longissimus muscle from beef, lamb, and pork carcasses. J. Anim. Sci..

[7] McCormick R.J. (1989). The influence of nutrition on collagen metabolism and stability. Recipr. Meat Conf. Proc..

[8] Scramlin S.M., Platter W.J., Gomez R.A., Choat W.T., McKeith F.K., Killefer J. (2010). Comparative effects of ractopamine hydrochloride and zilpaterol hydrochloride on growth performance, carcass traits, and longissimus tenderness of finishing steers. J. Anim. Sci..

[9] Rathmann R.J., Bernhard B.C., Swingle R.S., Lawrence T.E., Nichols W.T., Yates D.A., Hutcheson J.P., Streeter M.N., Brooks J.C., Miller M.F. (2012). Effects of zilpaterol hydrochloride and days on the finishing diet on feedlot performance, carcass characteristics, and tenderness in beef heifers. J. Anim. Sci..

[10] Rathmann R.J., Mehaffey J.M., Baxa T.J., Nichols W.T., Yates D.A., Hutcheson J.P., Brooks J.C., Johnson B.J., Miller M.F. (2009). Effects of duration of zilpaterol hydrochloride and days on the finishing diet on carcass cutability, composition, tenderness, and skeletal muscle gene expression in feedlot steers. J. Anim. Sci..

[11] United States Department of Agriculture—Agriculture Marketing Service (USDA-AMS) United States Standards for Grades of Carcass Beef. Agricultural Marketing Service. Washington, D.C. 2017. https://www.ams.usda.gov/sites/default/files/media/CarcassBeefStandard.pdf.

[12] Mohrhauser D.A., Taylor A.R., Underwood K.R., Pritchard R.H., Wertz-Lutz A.E., Blair A.D. (2015). The influence of maternal energy status during mid-gestation on beef offspring carcass characteristics and meat quality. J. Anim. Sci..

[13] American Meat Science Association (AMSA) (2015). Research Guidelines for Cookery, Sensory Evaluation, and Instrumental Tenderness Measurements of Meat.

[14] Hill F. (1966). The solubility of intramuscular collagen in meat animals of various ages. J. Food. Sci..

[15] Gerrard D.E., Jones S.J., Aberle E.D., Lemenager R.P., Diekman M.A., Judge M.D. (1987). Collagen stability, testosterone secretion and meat tenderness in growing bulls and steers. J. Anim. Sci..

[16] Mohrhauser D.A., Underwood K.R., Weaver A.D. (2011). In vitro degradation of bovine myofibrils is caused by μ–calpain, not caspase-3. J. Anim. Sci..

[17] Mersmann H.J. (1998). Overview of the effects of b-adrenergic receptor agonists on animal growth including mechanisms of action. J. Anim. Sci..

[18] Beckett J.L., Delmore R.J., Duff G.C., Yates D.A., Allen D.M., Lawrence T.E., Elam N. (2009). Effects of zilpaterol hydrochloride on growth rates, feed conversion, and carcass traits in calf-fed Holstein steers. J. Anim. Sci..

[19] McEvers T.J., May N.D., Reed J.A., Walter L.J., Hutcheson J.P., Lawrence T.E. (2018). The effect of zilpaterol hydrochloride on beef producer and processor revenue of calf-fed Holstein steers. Trans. Anim. Sci..

[20] Gruber S.L., Tatum J.D., Engle T.E., Mitchell M.A., Laudert S.B., Schroeder A.L., Platter W.J. (2007). Effects of ractopamine supplementation on growth performance and carcass characteristics of feedlot steers differing in biological type. J. Anim. Sci..

[21] McEvers T.J., Nichols W.T., Hutcheson J.P., Edmonds M.D., Lawrence T.E. (2012). Feeding performance, carcass characteristics, and tenderness attributes of steers sorted by the Igenity tenderness panel and fed zilpaterol hydrochloride. J. Anim. Sci..

[22] Gruber S.L., Tatum J.D., Engle T.E., Prusa K.J., Laudert S.B., Schroeder A.L., Platter W.J. (2008). Effects of ractopamine supplementation and postmortem aging on longissimus muscle palatability of beef steers differing in biological type. J. Anim. Sci..

[23] Avendaño-Reyes L., Torres-Rodríguez V., Meraz-Murillo F.J., Pérez-Linares C., Figueroa-Saavedra F., Robinson P.H. (2006). Effects of two β-adrenergic agonists on finishing performance, carcass characteristics, and meat quality of feedlot steers. J. Anim. Sci..

[24] Van Bibber-Krueger C.L., Miller K.A., Amachawadi R.G., Scott H.M., Gonzalez J.M., Drouillard J.S. (2017). Interaction between supplemental zinc oxide and zilpaterol hydrochloride on growth performance, carcass traits, and blood metabolites in feedlot steers. J. Anim. Sci..

[25] Bloomberg B.D., Mafi G.G., Pye B.J., Wahrmund J.L., Richards C.J., Morgan J.B., VanOverbeke D.L. (2013). Impact of health management, health treatments, and zilpaterol hydrochloride supplementation on carcass quality, color, and palatability traits in heifers. J. Anim. Sci..

[26] Cônsolo N.R.B., Ferrari V.B., Mesquita L.G., Goulart R.S., Silva L.F. (2016). Zilpaterol hydrochloride improves feed efficiency and changes body composition in nonimplanted Nellore heifers. Meat Sci..

[27] Garmyn A.J., Brooks J.C., Hodgen J.M., Nichols W.T., Hutcheson J.P., Rathmann R.J., Miller M.F. (2014). Comparative effects of supplementing beef steers with zilpaterol hydrochloride, ractopamine hydrochloride, or no beta agonist on strip loin composition, raw and cooked color properties, shear force, and consumer assessment of steaks aged for fourteen or twenty-one days postmortem. J. Anim. Sci..

